# Impact of Zika and Chikungunya Viruses on Spontaneous Abortions: Insights from a Reference Maternity Hospital

**DOI:** 10.3390/microorganisms13030678

**Published:** 2025-03-18

**Authors:** Anne Kerollen Pinheiro de Carvalho, Ana Cecília Ribeiro Cruz, Juarez Antônio Simões Quaresma, Arnaldo Jorge Martins Filho, Darlene de Brito Simith Durans, Orlando Pereira Amador Neto, Ligia do Socorro Oliveira de Lima, Norma Suely de Carvalho Fonseca Assunçao, Edna Cristina Santos Franco, Patrícia Brazão Cohen, Eliana Vieira Pinto da Silva

**Affiliations:** 1 Instituto Evandro Chagas, Seção de Arbovirologia e Febres Hemorrágicas, Ananindeua 67030-000, PA, Brazil; annekerollenef2@hotmail.com (A.K.P.d.C.); anacecilia@iec.gov.br (A.C.R.C.); darlenesimith@iec.gov.br (D.d.B.S.D.); 2 Department of Pathology, State University of Pará, Belém 66075-110, PA, Brazil; juarez@ufpa.br (J.A.S.Q.); brazaocohen@yahoo.com.br (P.B.C.); 3 Instituto Evandro Chagas, Seção de Patologia, Ananindeua 67140-000, PA, Brazil; arnaldofilho@iec.gov.br (A.J.M.F.); orlandoneto@iec.gov.br (O.P.A.N.); ligialima@iec.gov.br (L.d.S.O.d.L.); ednafranco@iec.gov.br (E.C.S.F.); 4 Fundação Santa Casa de Misericórdia do Pará, Belém 66030-010, PA, Brazil; normaassuncao@santacasa.pa.gob.br

**Keywords:** spontaneous abortion, placental detection, Zika, Chikungunya

## Abstract

To investigate the association between miscarriage and ZIKV and CHIKV infection. The study population consisted of pregnant women who had miscarriages between 2015, 2016 and 2017, comprising a total of 30 women who were treated at the Santa Casa de Misericórdia do Pará Foundation (FSCMPA). The processed samples came from already paraffinized material containing placental and fetal remains, where they were tested with hematoxylin–eosin and immunohistochemistry for ZIKV and CHIKV. Regarding the sociodemographic, clinical and obstetric characteristics of the patients, they correspond to the age group between 20 and 29 years of age; of brown color; women who had abortions for the first time; miscarriages occurring in the first trimester of pregnancy; women belonging to the metropolitan region of Belém; diagnosed with incomplete abortion and who had undergone uterine curettage procedure. Regarding the histopathologic and immunohistochemical findings, an inflammatory infiltrate rich in neutrophils and lymphocytes, among others, was found in the endometrial fragments and chorionic membranes. In addition, placental areas consisting of edema, necrosis and hemorrhage were found. The study identified ZIKV and CHIKV in 40% (n = 12) of samples from spontaneous abortion specimens, and CHIKV was the most prevalent virus in the study, representing 36.11% of the total specimens, with reddish granular material in the cytoplasm of decidua cells and placental villi suggesting that the viruses may be present in these regions of the placenta.

## 1. Introduction

The World Health Organization (WHO) defines abortion as the involuntary termination of pregnancy up to 22 weeks’ gestation, which, in quantified terms, would correspond to a fetal weight of approximately 500 g [1]. Spontaneous abortion affects a large number of women, with most occurring in the first 12 weeks of pregnancy [2].

It is estimated that 25% of miscarriages could be prevented if risk factors could be reduced. However, approximately 50% of miscarriages have unknown causes [3].

Studies have shown that viruses such as cytomegalovirus (CMV), herpes simplex virus (HSV-1/2), human papillomavirus B19 (B19V), enterovirus, adenovirus, and varicella zoster virus are the most common causes of miscarriage [4,5,6,7]. However, other causes may be associated with miscarriage.

The reported consequences of maternal infection caused by some arboviruses include placental transmission, miscarriage, congenital malformations, stillbirths, intrauterine growth restriction, and preterm delivery [8].

In recent years, sone evidence has led to the belief that arboviruses are present in the maternal and child context and should be monitored, seeking preventive strategies and better clinical management for pregnant women and their concept, or neonate, who may become ill [9,10,11].

Arboviruses are characterized by a group of viral diseases, transmitted by vectors (arthropod-borne viruses) to susceptible vertebrate hosts. Arboviruses are viruses transmitted to humans by the bite of hematophagous arthropod vectors, as part of the viral replicative cycle that occurs in these arthropods [12].

Currently, the most prevalent viruses in Brazil are the *Orthoflavivirus denguei* (DENV), *Chikungunya virus* (CHIKV) and the *Orthoflavivirus zikaense* (ZIKV) [13]. DENV and ZIKV belong to the *Flaviviridae* family, while CHIKV belongs to the *Togaviridae* family [14].

For fetal infection by ZIKV to occur and cause fetal malformations, the highest probability occurs during the first trimester of pregnancy, in which the trophoblast is more permissive to ZIKV, presenting immaturity of the villi, reducing its defenses in controlling infections, causing the virus to attack fetal neural tissue, causing neurodevelopmental abnormalities. Spontaneous abortions, fetal losses and neonatal deaths have also been observed. These occurrences are related to the gestational period in which there was exposure to the virus [15].

Infection with ZIKV has been identified as the cause of spontaneous abortion, taking into account the vertical transmission from the positive mother during pregnancy [16].

Another arbovirus associated with maternal–infant complications is CHIKV, as there is evidence of a high risk of abortion in the first trimester and maternal–fetal transmission in the last trimester [13,17].

Among the possible obstetric complications related to CHIKV are spontaneous abortion in the first trimester, pre-eclampsia in the second and third historical trimesters and hospitalization in an intensive care unit (ICU), due to viral sepsis caused by CHIKV. Among the obstetric pathologies, premature rupture of membranes, intrauterine growth restriction, pre-eclampsia, preterm delivery and postpartum hemorrhage have been reported. Among pregnant women infected with CHIKV, (15%) required admission to an ICU and (89%) were in the third trimester of pregnancy [18].

It should be noted that there is still no effective treatment that can be used safely in pregnant women who are affected by such arboviruses. Research is underway, trying to obtain better results in reducing the damage caused by the triple viral infection in an attempt to reduce its effects as well as to alleviate the complications for the pregnant woman and her fetus [19].

Conducting epidemiologic and clinical research to improve the knowledge for the prevention of infection and improve care for women who are affected by spontaneous abortion is the basis of this study. Now, it is known that many women are assisted in hospitals after having an abortion; however, most of the time, it is not known why this abortion happened.

It is necessary to better investigate the etiology of these spontaneous abortions, especially if there is a correlation with ZIKV and CHIKV infection, as they may be capable of causing abortion.

Therefore, this study aims to investigate the association of spontaneous abortions with the occurrence of infections by the Zika virus and Chikungunya virus.

## 2. Methods

### 2.1. Study Design and Sampling

This is a descriptive, longitudinal, retrospective, qualitative–quantitative study. According to Carlos (2017), descriptive research is used when it aims to describe the characteristics of certain populations or phenomena [20]. The longitudinal study is intended to study a process over time to examine changes, that is, reflect a sequence of facts. These are studies that investigate the prevalence of new cases of a particular disease in a population, and can be conducted at different time intervals [21]. In the case of a retrospective study, the researcher can collect past information on the exposure factor(s) (hence the term retrospective) and follow the subjects over a period of time (the cohort) [22].

The sample universe consisted of pregnant women who had a miscarriage in the years 2015, 2016 and 2017, comprising an “N” sample of 30 women who received care at the Fundação Santa Casa de Misericórdia do Pará (FSCMPA). The study material comprises tissue block samples in paraffin containing material from the miscarriage, corresponding to placental and/or fetal remains.

The study period was chosen because the arboviruses under investigation were circulating at the same time. Women who had a spontaneous abortion, with a gestational age up to 22 weeks, or the product of the abortion weighed less than 500 g, without a previously known cause, who underwent curettage or MVA procedures and who were examined for histopathologic evaluation, were included in the research. Corresponding to the samples in paraffin blocks, these were sent to the Ruth Brazão Laboratory.

Pregnant women who underwent curettage or MVA procedures and examination collection for histopathologic evaluation were excluded from the study, and these were sent to another laboratory other than the Ruth Brazão Laboratory and also pregnant women who did not have suspicious or suggestive symptoms of ZIKV infection and CHIKV, such as fever, headache, retro-orbital pain, muscle and/or joint pain, among others.

### 2.2. Data Collection

**Figure 1:**
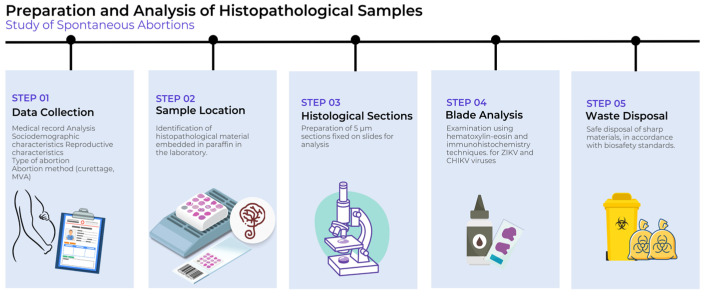
Flowchart representing the stages in the process of preparing and analyzing histopathological samples in the study of miscarriage: 1. Data collection, 2. Location of histopathological material, 3. Sample preparation, 4. Laboratory examination, 5. Disposal of sharps.

The data collected consisted of the following five steps:

(a) Step 1—The data collection was carried out through the analysis of medical records, during the aforementioned period, by filling in a pre-prepared, which was used to organize the data and included the following study variables: sociodemographic characteristics (origin, age group, race and marital status); reproductive characteristics (pregnancy history, parity history, abortion history, gestational age at death); clinical conditions (symptoms); type of abortion (threatened abortion, complete abortion, incomplete abortion, missed abortion, and habitual or recurrent abortion); method of abortion (curettage, MVA). The women who were included in the study, according to the inclusion criteria, had their medical records filled in, as well as the order number of the histopathological examination, so that they could be identified in the external laboratory (Ruth Brazão Laboratory), associated with the FSCMPA, to which the collection of histopathological material was sent.

(b) Step 2—The histopathologic material, already embedded in paraffin, was located in the aforementioned laboratory according to the numbering of the histopathologic examination work order contained in the medical record of each selected patient, according to the inclusion criteria.

(c) Step 3—In the pathology laboratory of the IEC, 5 μm sections were made from the paraffin blocks of each selected patient, which were fixed on 8 different slides for the immunohistopathological analyses. The histologic sections obtained were stained and examined by optical microscopy in the aforementioned laboratory. The remainder of the blocked material was returned to the laboratory, in order to preserve the sample.

(d) Step 4—The 8 slides prepared from each patient were examined by the techniques of hematoxylin–eosin and immunohistochemistry for ZIKV and CHIKV, one of which was kept in reserve, in case the experiment needed to be repeated.

(e) Step 5—After completion of the steps, the resulting waste, corresponding to the sharps was properly disposed in a sharps collector (discarded) according to the biosafety criteria contained in RDC No. 222, dated 28 March 2018, which regulates the good practices of waste management in health care, among other provisions. A summary of these steps can be seen in Figure 1.

### 2.3. Histopathology

After fixation of the tissue samples obtained from curettage or MVA, they were processed for histopathologic analysis according to the routine hematoxylin–eosin (HE) staining protocol of the pathology laboratory. First, the samples were dehydrated in graded baths of 70% to 100% alcohol for one hour and twenty minutes each step, followed by two immersions in a xylene bath at room temperature and successive baths in liquid paraffin, maintained at a temperature of 60 °C. The material was then embedded to form paraffin blocks. In this study, the paraffin blocks were already prepared and located to perform the microtomy of the samples in 5 μm sections, performed in a LEICA histotechnician TP 1020 (Leica Biosystems, Nussloch, Germany). The histological sections were then stained by the HE technique and examined under an optical microscope.

### 2.4. Immunohistochemistry (IHC)

For the detection of viral antigens of ZIKV and CHIKV in histological sections, the immunohistochemistry technique was performed [23]. The 5 μm sections of the blocks were harvested on slides previously prepared with 3-amino-propyltriethoxy-silane adhesive solution (Sigma, San Luis, MO, USA). Then this material was deparaffinized in two xylene baths at room temperature for 5 min each and then rehydrated in decreasing sequence with absolute alcohol (100%, 95% and 70%) for five minutes each and distilled water.

Subsequently, antigen retrieval was performed, placing the slides on a plastic support and adding EDTA (Sigma, San Luis, MO, USA) inside the metallic cuvettes until the tissue was completely covered. Then the metallic cuvettes were placed in 500 mL of distilled water in an electric pressure cooker for 15 min at a temperature of 110 °C. After removing the cuvettes from the pressure cooker, a jet of distilled water was poured over them. The tissue was incubated with nonspecific reaction blocking solution (background punisher) for 10 min at room temperature. In the next step, the samples were incubated with the polyclonal antibody produced “in house” in adult mice by the intraperitoneal route against the aforementioned viruses and incubated for 30 min at room temperature, with a titration of 1:200.

Slides were washed twice with TBS (TRIS Saline Buffer) (Sigma, San Luis, MO, USA) solution. Subsequently, the tissue was incubated with secondary antibody from the commercial kit (M4CH4 Universal AP-Prob) (Termo Fisher Scientific^®^ Waltham, MA, USA) for 10 min at room temperature. The slides were washed with TBS solution and incubated with enzymatic polymer for 15 min and then washed twice more with TBS solution. The reaction was developed with chromogen solution (Fast Red) (Sigma, San Luis, MO, USA) for 20 min at room temperature, then washed with distilled water. For counterstaining, hematoxylin was added for 2 min, then washed with distilled water for 1 min. Slides were mounted using Permount resin (Termo Fisher Scientific^®^ Waltham, MA, USA).

Photo documentation was performed by means of a photographic record with a camera attached to the Axio autoimage Z2 microscope (Zeiss, Oberkochen, Germany), at 400× magnification of the spontaneous abortion samples.

### 2.5. Statistical Analysis

The data obtained during the study were stored in Microsoft Office Excel (2010) Versão 2007 spreadsheets. The method used to analyze the quantitative data obtained from the medical records is based on descriptive statistics through absolute and relative frequencies, seeking to verify if the data converge to a particular differential or if there is a tendency in their distribution. Comparisons between the distributions of the variables were performed using the Chi-square test for tendency/adherence, which is a hypothesis test designed to verify whether or not there is a tendency in the distribution of nominal and ordinal variables [24], adopting a significance level of *p*-value < 0.05. Statistical analyses were carried out using the Bioestat 5.4 software.

### 2.6. Ethical Statement

The present study was developed in the Pathology Department of the Evandro Chagas Institute (IEC) in collaboration with the Santa Casa de Misericórdia do Pará Foundation (FSCMP) and Ruth Brazão Laboratory. It began after approval by the IEC’s Committee for Ethics in Human Research (CEP) and the CEP of the FSCMPA. The study was submitted for review and submission according to Resolution 466 of 2012 of the National Health Council of the Ministry of Health, approved by the Certificate of Ethical Assessment (CAAE) under the numbers 18613819.3.0000.0019 and 18613819.3.3001.5171.

## 3. Results

### 3.1. Clinical and Obstetric Sociodemographic Profile

Table 1 shows the sociodemographic characteristics of women who had spontaneous abortions and were treated at the FSCMPA. It can be seen that the highest significant frequencies were: 53.33% (n = 16) of women aged 20–29 years, coming from the state capital (Belém) corresponding to 70% (n = 21), the majority of women, who declared their skin color, reported being brown, 66.67% (n = 20). Regarding marital status, those who provided the information in the medical record corresponded to the majority of single women 36.67% (n = 20), which showed a certain tendency in terms of significance.

Table 2 shows the conditions of pregnancy, type and performance of abortions of women who had spontaneous abortions treated at the FSCMPA. It is observed that there is a predominance of symptoms of headache 23.34% (n = 7) and fever 20% (n = 6) during the hospitalization of patients who had miscarriage, and that 43.33% (n = 13) did not report any symptoms. Of the women, 33.33% (n = 10) were in their second pregnancy when they had an abortion, 36.67% (n = 11) already had one child, as they already had a history of parity; these data were close to the significance value. The statistically significant variables were related to the majority of women, 66.67% (n = 20), who were having an abortion for the first time. Sixty percent (n = 18) of the women were in the first trimester of pregnancy, most in the 12th week, when they had a miscarriage; 80% (n = 24) were classified as incomplete abortion and 86.67% (n = 26) had the curettage in the abortion.

### 3.2. Aspects of Histopathology and Immunohistochemistry

**Figure 2:**
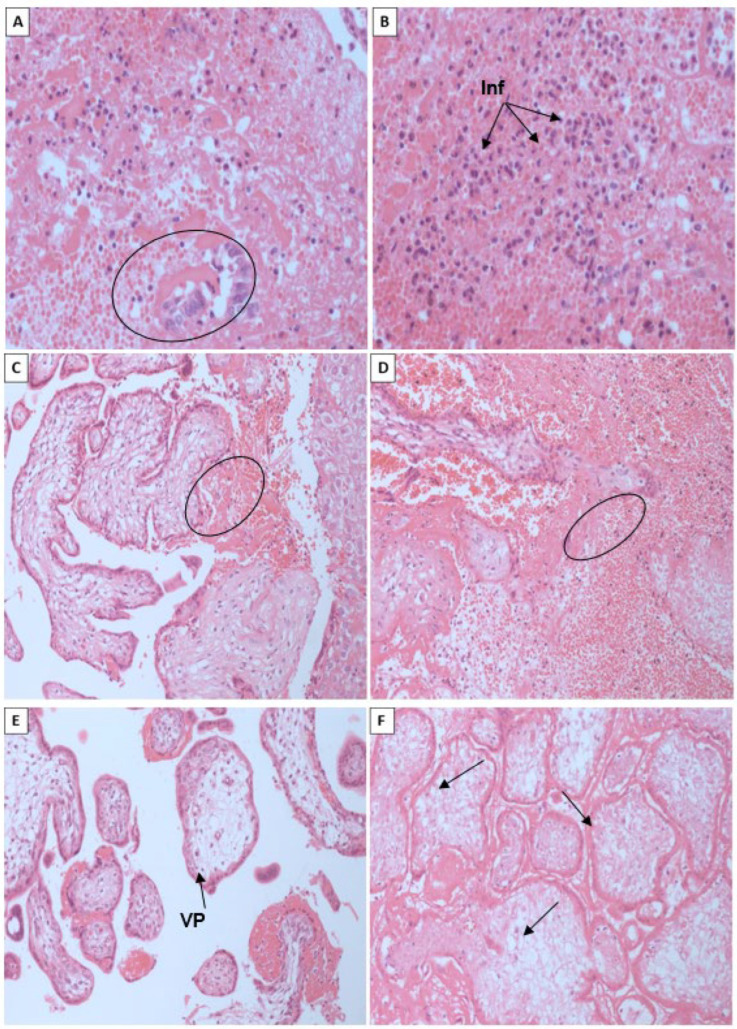
Histopathological analysis of fetal material and gestational tissues from the product of abortion: (**A**) Arias-Stella phenomenon (AS) highlighted within the circles; (**B**) Inflammatory infiltrate (Inf) of mononuclear lymphocytes, plasma cells and neutrophils, identified by arrows; (**C**,**D**) Hemorrhage demarcated within the circles; (**E**) Placental villi (PV) showing a delicate swollen conjunctival mesh, indicated by the arrow; (**F**) Tissue necrosis demonstrated by the arrow. Staining: Hematoxylin-eosin. Magnification 400×. Source: Research protocol, 2022.

In all the samples studied, the findings of the uterine contents were common, as shown in Figure 2, consisting of (A) fragments of endometrium with a gravid secretory pattern in which glandular structures were lined with columnar epithelium with abundant cytoplasm, sometimes clear and homogeneous, often showing secretion by decapitation of the apical portion of the epithelium (Arias-Stella phenomenon—dark circle). The cell nuclei were basalized and normochromatic, surrounded by a congested stroma and permeated by an inflammatory infiltrate of mononuclear lymphocytes and plasma cells, associated with moderate neutrophils.

We also observed representative fragments of decidua permeated by inflammatory infiltrate rich in neutrophils, in the midst of blood clots, hemorrhage (dark circles in Figure 2C,D), and necrotic tissue fragments.

Regarding the placenta, there is a representation of placental villi (Figure 2E, dark arrow) covered by two different cell layers corresponding to syncytiotrophoblast (STB) and cytotrophoblast (CTB), respectively, with a central stroma consisting of a delicate and swollen conjunctival mesh.

In some cases, chorioamnion membranes consisting of elongated cells were present, permeated by a mixed inflammatory infiltrate of neutrophils and lymphocytes.

The extensive associated necrosis (black arrows in Figure 2F), as well as the areas represented by blood clots, were very often associated with the extensive inflammatory component in these areas, consisting of lymphocytes, plasma cells, neutrophils and macrophages.

**Figure 3:**
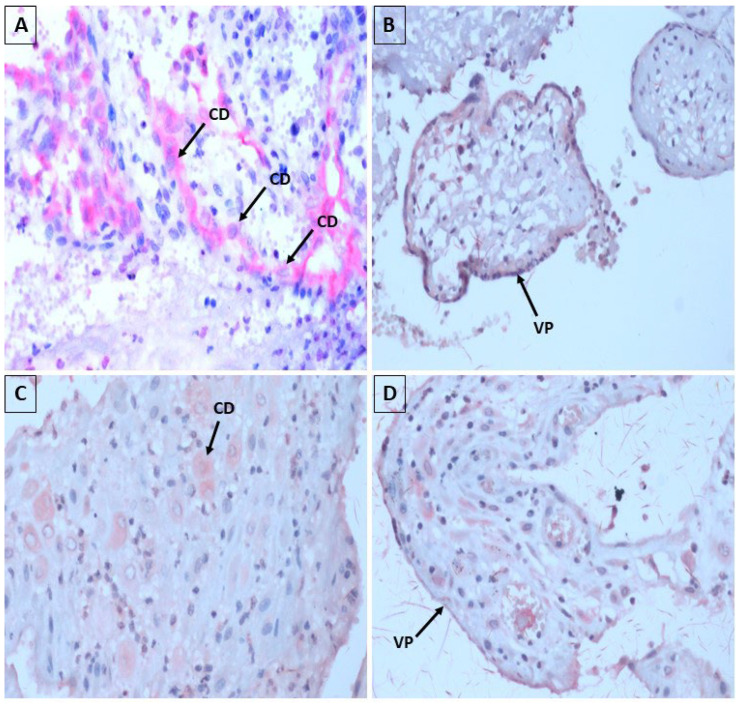
Detection of ZIKV and CHIKV virus antigens in samples of spontaneous abortion using the immunohistochemical technique. Source: Research protocol, 2022. NOTE: (**A**,**B**) Detection of ZIKV in decidual cells (CD) and placental villi (VP). (**C**,**D**) Detection of CHIKV in decidual cells (DC) and placental villi (VP). Magnification: 400×.

The immunohistochemical evaluation of these samples revealed a focal positivity, represented by the deposition of reddish granular material in the cytoplasm of decidua cells and placental villi, both for antibodies against ZIKV and antibodies against CHIKV (Figure 3).

Thus, of the 30 samples, 26 were analyzed for ZIKV and CHIKV and 4 samples were analyzed for CHIKV only, corresponding to three samples from 2015 and one sample from 2017. The four samples analyzed for CHIKV only were the last samples to be processed, and due to the pandemic, it was difficult to continue the study to process them for ZIKV.

Table 3 shows the detection of the occurrence of CHIKV and ZIKV in the years 2015, 2016 and 2017 among women who had spontaneous abortions in the FSCMPA.

Focal positivity of ZIKV and CHIKV by immunohistochemical analysis was found in 40% (n = 12) of the samples in our study. Three samples were positive for both ZIKV and CHIKV, 3.33% (n = 1) for the year 2015 and 6.66% (n = 2) for the year 2017. The statistically significant data were for the year 2015, in which 11.11% (n= 3) were positive for CHIKV and 7.40% (n = 2) positive for ZIKV. In the year 2016, all samples were negative for virus. In the 2017 samples, 25% (n = 7) were positive for CHIKV and 10.71% (n = 3) were positive for ZIKV.

## 4. Discussion

Spontaneous abortions are more common in high-risk pregnancies, especially in women who are not receiving adequate medical care. It is imperative to raise awareness among health professionals so that they can correctly and thoroughly investigate these situations, so that the causes and risk factors can be identified to prevent future fetal damage. This study will facilitate the understanding of one of the possible risk factors that may be associated with this during infections of pregnant women by these arboviruses [25]. In relation to the sociodemographic, clinical and obstetric data portrayed in the study, abortion generally occurs in the first trimester of pregnancy [26]. Therefore, understanding its causes could be an important aspect of prevention. It is estimated that 25% of miscarriages could be avoided if the risk factors could be reduced [1]. However, around 50% of these cases have unknown causes. They may or may not be related to genetic factors.

Non-genetic causes include sociodemographic factors associated with advanced maternal age (>35 years). This high maternal age, greater than 35 years, has been considered by some authors to be a risk factor for miscarriage, as well as fetal malformations, due to the senility of the oocyte, which is more prone to chromosomal alterations in cases of fertilization [27]. Every 5-year increase in maternal age increases the risk of miscarriage by 1.5 times [3,28,29].

However, some studies found a contrary association. One explanation for this divergence lies in the fact that studies that associated maternal age over 35 years with abortion were carried out in developed countries, where women are likely to become pregnant at an older age; and studies that address maternal age below 35 years as a risk factor were carried out in developing countries, where women become pregnant earlier, increasing the likelihood of miscarriage [30,31,32]. This corroborates the data of our study, in which women aged between 20 and 29 years predominated.

Our study showed that the women who had abortions at the FSCMPA came from the state capital (Belém). This high-risk referral maternity hospital receives a large number of pregnant women on spontaneous demand, referred from the metropolitan region of Belém and from municipalities in the interior of the state of Pará. A study by Durex et al. (2016) shows the number of hospital admissions for abortion in the regions of Brazil between 2010 and 2014 in the North region, with 5.79 admissions per 1000 women of childbearing age (10–49 years) [33]. The scarcity of studies of this nature involving the northern region of Brazil is notorious.

In our study, most of the women said they were brown. According to studies by Soares and Cançado (2018), carried out at the Santa Casa de Misericórdia de Passos, in Minas Gerais (MG), the majority of women who had a miscarriage, according to ethnicity, 47.5% were white, unlike our results. However, the highest prevalence of miscarriage was observed in brown women (66%), followed by white women (28%) and black women (6%) [34], corroborating our results. Thus, these studies show that ethnicity may indeed differ according to the population group studied.

Another fact highlighted in this study was the absence of a steady partner in 44% of the women who suffered a miscarriage [35]. This data corroborates our research, in which the majority of these women were single. This can be explained by the lack of emotional support and the economic instability to which women who do not have a stable marital relationship are subject [36].

Most infections caused by arboviruses are asymptomatic, but 20% to 25% of these people have nonspecific clinical manifestations, giving rise to the need for a differential laboratory diagnosis between CHIKV and DENV [36]. ZIKV or CHIKV infection should be suspected if two or more of the following symptoms occur: absence or low fever ≤ 38 °C (1–2 days of low fever) in ZIKV and high fever > 38 °C (2–3 days) in CHIKV, skin rashes, muscle pain, joint pain, conjunctivitis, headache, among others. In our study, the majority of women presented with headache followed by fever, ranging from 37 to 38 °C, at the time of admission for abortion.

In this study, the majority of women who had miscarriages for the first time had already had a previous pregnancy, which corroborates the study by Soares and Cançado (2018) [34] where, in the previous history of these patients, 82.5% had a pregnancy loss during their first pregnancy and 60% had had a previous pregnancy. Data evidenced in the literature that there is a higher rate of abortions, with a greater number of children [37,38].

In our study, the majority of women had uterine curettage as an abortion procedure. A study on therapeutic approaches in the uterine abortion process identified that uterine curettage was the second most observed treatment, accounting for 175 (37.55%) indications for this therapy. MVA was responsible for 85 (18.24%) indications for uterine evacuation, although 370 (79.40%) patients had early miscarriage [39,40].

It can be concluded that the obstetric profile of women who experience pregnancy loss does not differ from that found nationally; curettage was the most used final therapy, plus some unfavorable outcomes, such as longer hospital stay and excessive exposure to medication [39,41].

It is possible to show that MVA is a good alternative in the management of first trimester abortion due to its lower costs, fewer complications, high efficiency in the resolution of the pathology and great satisfaction on the part of users. According to international organizations, the use of uterine curettage should be eliminated from obstetric practice, replacing it with manual or electric aspiration, or the use of medication [42,43].

However, deciding on the best conduct in the treatment of miscarriage is still a major challenge for prescribers today, reflecting the lack of information based on scientific evidence that allows for safe and effective conduct [44].

Our study showed that most women had incomplete abortion. In abortion, the main conduct was incomplete abortion, accounting for 44.85% (n = 209) of hospitalizations for uterine emptying, followed by fetal death, with 34.98% (n = 163). Regarding the treatment of choice, misoprostol was the most used, at 59.01%, followed by the curettage procedure 37.55%, both used associated or not [45].

Regarding the histopathological and immunohistochemical aspects evidenced, the vertical transmission of arboviruses that may have caused spontaneous abortions, described in our research, has already been described in other arboviruses. Vertical transmission of arboviruses, caused by flaviviruses including West Nile, Dengue and Zika, where they found viral replication in the placenta of humans and mice [46,47].

The pathogenesis of the process of transplacental transmission of ZIKV, although well accepted in the literature, is still poorly understood [48]. The detection of the CHIKV genome in the placentas of pregnant women with virus infections has already been reported in the literature previously [49,50,51,52]. The association between ZIKV infection and miscarriage has also been demonstrated in a study with non-human primates [53].

In our research, with the use of immunohistochemistry, in 40% of the samples, the antibodies against ZIKV and antibodies against CHIKV revealed focal positivity, with predominance for CHIKV, with reddish granular material in the cytoplasm of the decidua cells and placental villi, elucidating that the virus may be present in these placental regions, which was also evidenced in the study by Annemiek et al., 2022 [54] in which in situ hybridization using ZIKV-specific probes, compared to control probes, revealed that placental amniotic epithelium was positive for ZIKV RNA, while fetal chorion and trophoblasts and maternal decidua were not showing evidence of ZIKV infection [55].

It was possible to observe placental villi coated with STB and CTB containing swollen conjunctival mesh in the spontaneous abortion samples, suggesting that ZIKV seems to be able to induce vascular damage followed by apoptosis in the placental tissue, making the placenta more permeable, and facilitating the entry of the virus into syncytiotrophoblastic cells. Once in the placental tissue, ZIKV can replicate in other cell types, such as macrophages and fetal endothelial cells, acting as true deposits of the virus, allowing its dissemination in fetal blood [55].

Using immunohistochemical techniques, the CHIKV antigen was detected in the epithelial cells of the endometrial glands and in the decidual cells, in the case of spontaneous abortion. In addition, CHIKV antigen was also detected in some chorionic villus STB. Likewise, our findings corroborate those of the aforementioned author [56].

STBs are also an important cell type that undertakes the endocrine function of the human placenta to drive the physiological and metabolic adaptations to pregnancy. CTBs have a high proliferation capacity and form a monolayer of polarized stem cells, which eventually differentiate via cell–cell fusion into STBs that cover the entire surface [57].

Different subtypes of trophoblasts have different susceptibilities to pathogenic infections. Although the mechanisms are still poorly understood, STBs have been shown to be resistant to infection by several pathogens, other than the arboviruses reported in our study. In the recent pandemic, it was proposed that SARS-CoV-2 also prefers to target STB, probably due to the high abundance of viral receptors on its apical surface [57].

It is worth mentioning that syncytiotrophoblastic cells produce human chorionic gonadotropin (hCG), a very important hormone needed to stimulate the corpus luteum in the continuous production of progesterone, which in turn is necessary to maintain a pregnancy. Once infected, production can be altered, leading to miscarriage. Perhaps, the involvement of the STBs impaired the production of hCG, which possibly culminated in the fatal outcome of the spontaneous abortion cases reported in our study [58,59].

A study conducted by Martines et al., 2016 [59] described two cases of miscarriage due to the ZIKV. One of the reported cases was a previously healthy 37-year-old woman who developed fever and rash at 8 weeks’ gestation and subsequently miscarried at 11 weeks’ gestation. It is important to mention that no drug or chemical exposures were reported. Prenatal serologic testing was negative for cytomegalovirus, rubella virus, toxoplasma gondii, herpes simplex virus and HIV. Placental blocks were available for evaluation. Placental tissue showed dense and heterogeneous chorionic villi with calcification, sclerosis, edema, increased perivillous fibrin deposition and irregular lympho-histiocytic intervillous inflammation. Immunohistochemistry of placental tissue was positive for Zika virus, with antigens observed on Hofbauer cells in the chorionic villi [59].

In another study carried out on Réunion Island, vertical transmission of CHIKV was first observed in June 2005 during the CHIKV epidemic that affected more than one-third of the local population from March 2005 to July 2006. However, evidence of transplacental infection was reported following the recovery of the CHIKV viral genome from amniotic fluid, placenta and fetal brain of stillborn fetuses on Réunion Island when fetal loss occurred at less than 16 weeks of gestational age. The maternal diagnosis of chikungunya was confirmed by RT-PCR detection in maternal blood two weeks before the diagnosis of fetal loss; these detections of viral genome in placenta and amniotic fluid confirmed transplacental transmission (vertical transmission) of CHIKV as well as its persistence after fetal death, demonstrating once again the viral tropism of arboviruses for placental tissues [49].

The other case was a previously healthy 31-year-old woman who presented with fever and rash at 8 weeks gestation. No drug or chemical exposures were reported. She had miscarried at 13 weeks’ gestation. Ultrasound at 6 weeks’ gestation showed no abnormalities, and prenatal serologic tests for HIV, herpes simplex virus, hepatitis B and C viruses, cytomegalovirus (IgM), rubella virus (IgM), and *Toxoplasma gondii* were negative. Paraffin blocks from the curettage specimen were available for examination [59].

The specimens showed predominantly endometrial tissue with Arias-Stella reaction. Tiny pieces of placental tissue showed no significant findings. Immunohistochemistry was negative for ZIKV. ZIKV RT-PCR was positive and sequence analysis showed 100% identity with ZIKV strains isolated in Brazil in 2015 [55]. The Arias-Stella reaction observed in the aforementioned study was also found in fragments of endometrium in our study, often showing secretion by decapitation of the apical portion of the epithelium.

We also found intense areas of inflammatory infiltrate in fragments of the decidua, forming areas rich in neutrophils, necrotic tissue and hemorrhages. In the chorioamnionic membranes, the cells showed inflammation with the presence of neutrophils and lymphocytes. In a study of a woman with suspected ZIKV infection who had a spontaneous abortion, in which amniocentesis was performed, followed by dilation and curettage, the histopathologic examination and immunohistochemistry using CD45 and CD3 antibodies to detect inflammation showed no evidence of increased infiltration of inflammatory cells [55]. This contradicts the findings of our study.

However, in another study in a pregnant woman diagnosed with incomplete abortion and with a positive serologic test for CHIKV, the histopathologic findings showed that there was an extensive area of inflammatory infiltrate in the decidua, composed mainly of lymphocytes and neutrophils, characterized by their morphology in infected tissue. The chorionic villi showed changes such as inflammatory infiltrate and areas of calcification, edema and deposition of fibrinoid material [56].

In the same study, another pregnant woman’s case was reported with a positive serologic test for CHIKV having a spontaneous abortion, in which curettage was performed. The chorionic villi also showed areas of fibrin deposition and edema, except for calcifications and inflammatory infiltrate. In addition, the intervillous space showed infiltrate of lymphocytes [56]. Therefore, the presence of inflammatory infiltrate may be a factor associated with abortion, but it is not a single factor and requires the presence of other factors.

In a report on spontaneous abortion, irregularities were observed in the chorionic villi. These irregularities included edema which is recognizable by open spaces in the cytoplasm of intervillous cells and in the interstitium of villi. This edema could be a possible cause of prenatal hypoxia [1]. It is noteworthy that the swelling observed in regions of the placental villus is also described in our study. Placental tissue from spontaneous abortion caused by ZIKV infection may exhibit features such as edema, deposition of fibrinoid material and calcification. Swollen conjunctive meshes may also be observed in cases of spontaneous abortion [60].

In our study, extensive associated necrosis was observed, and areas represented by blood clots were frequently associated with an extensive inflammatory component. The results of deaths of stillbirths and neonates caused by ZIKV infection showed an intrinsic and complex relationship between the immune response and the occurrence of cell damage. The main types of cell death found were necrosis and apoptosis [61].

There are few studies showing that CHIKV can cause long-term morbidity and death in fetuses and neonates infected during pregnancy [62] (We observed that three pregnant women with both the ZIKV and CHIKV coinfection culminated in spontaneous abortion. Coinfection of ZIKV and other arboviruses has been previously documented with: DENV and CHIKV in Colombia, DENV, ZIKV, CHIKV in some areas of Mexico and with DENV in French Polynesia and New Caledonia [1]. The magnitude of arbovirus co-infection during pregnancy is complex and is not yet clear, especially due to the high frequency of asymptomatic infections and cross reactions between flaviviruses, such as ZIKV and DENV. The impact of co-infection caused by arboviruses is little known, even though a fatal co-infection caused by DENV and CHIKV has already been reported in Colombia [63,64].

A study showed that a woman in her second trimester of pregnancy was diagnosed by RT-PCR with a co-infection caused by CHIKV and ZIKV after an abnormal ultrasound, with evidence of the absence of fetal heartbeat. After fetal autopsy, it was possible to observe low birth weight, along with renal and placental calcifications. Such findings are in agreement with our cases of co-infections reported in our study with ZIKV and CHIKV. It is worth mentioning that complementary studies such as detection of the viral genome by RT-PCR of the study samples need to be performed so that we can affirm these findings more strongly [52,65].

Thus, the infection possibly caused by arboviruses in the placenta, may cause placental dysfunction, leading to adverse effects on the fetus, making further research on this placental collection necessary. Additionally, the scarcity of updated regional studies on the subject, as well as maternal clinical data, needs to be clarified with the acquisition of new data on arboviruses that have already been associated or known in pregnancies, as well as the etiology of abortions in the metropolitan area of Belém and other regions of Pará state.

## 5. Conclusions

Our study detected the presence of ZIKV and CHIKV in the first trimester of pregnancy, which may be associated with damage, placental and fetal losses. A more in-depth evaluation of these abortions is necessary to understand the pathogenesis of these arboviruses. We identified ZIKV and CHIKV in 40% (n = 12) of our sample universe, with CHIKV being the most prevalent arbovirus in the study, corresponding to 36.11% of the total samples.

Our findings showed that the most statistically significant data refer to sociodemographic, clinical and obstetric characteristics, especially women aged between 20 and 29 who had their first abortion in the first trimester of pregnancy, women from the metropolitan region of Belém who underwent uterine curettage due to incomplete abortion. We analyzed the histopathological and immunohistochemical findings of endometrial fragments and chorionic membranes. The sample showed a contracted inflammatory infiltrate rich in neutrophils and lymphocytes, among other cells.

In addition, placental areas characterized by edema, necrosis, and hemorrhage were found. These findings are typical in all cases positive for CHIKV and ZIKV.

## Figures and Tables

**Table 1:** Sociodemographic characteristics of women who had a miscarriage in the FSCMPA.

Sociodemographic Profile	n	%	*p*-Value ^1^
Age group (in years)			
12–19	3	10.00	0.003 *
20–29	16	53.33	
30–39	10	33.33	
≥40	1	3.33	
Origin			
State capital	21	70.00	<0.001 *
Other metropolitan areas of Belém	5	16.67	
Municipalities in the interior of Pará	3	10.00	
Not mentioned	1	3.33	
Race			
White	3	10.00	
Brown	20	66.67	0.0004 *
Not mentioned	7	23.33	
Marital status			
Married	2	6.67	0.1116
Single	11	36.67	
Union stability	8	26.67	
Not mentioned	9	30.00	

Source: Research Protocol, 2022. ^1^ Chi-square adherence (*p*-value < 0.05). * Significant values. Note: Metropolitan Regions: Ananindeua, Benevides, Castanhal, Marituba and Santa Izabel do Pará. Municipalities in the interior of Pará: Abaetetuba, Acará, Aurora do Pará, Barcarena, Bonito, Bujaru, Cajoeira do Arari, Capanema, Colares, Concórdia do Pará, Maracanã, Moju, Ourém, Paragominas, Santa Cruz do Arari, Santa Maria do Pará, Santo Antônio do Tauá, São Domingos do Capim, Tomé-Açu, Vigia and Viseu. Regions belonging to the study.

**Table 2:** Analyzed parameters of women who had spontaneous abortion in the FSCMPA.

Parameters	n	%	*p*-Value ^1^
Symptoms			
Headache	7	23.34	0.0073
Fever	6	20.00	
Malaise	1	3.33	
Vomiting	3	10.00	
Not mentioned	13	43.33	
Pregnancy history			
1	3	10.00	
2	10	33.33	
3	8	26.67	0.2255
4	5	16.67	
≥5	4	13.33	
Parity history			
0	6	20.00	
1	11	36.67	
2	7	23.33	0.1046
3	4	13.33	
4	2	6.67	
Abortion history			
0	20	66.67	0.0004 *
1	6	20.00	
2	4	13.33	
Gestational age (weeks**)**			
1–13	18	60.00	<0.0001 *
14–28	10	33.33	
29–41	0	0.00	
not mentioned	2	6.67	
Type of abortion			
Incomplete abortion	24	80.00	0.0047 *
Missed abortion	6	20.00	
Abortion procedure			
Manual intrauterine aspiration	4	13.33	0.0004 *
Curettage	26	86.67	

Source: Research protocol, 2022. ^1^ Chi-square adherence (*p*-value < 0.05). * Significant values.

**Table 3:** Demonstration of the occurrence of CHIKV and ZIKV in the years 2015, 2016 and 2017 in women who had a spontaneous abortion in the FSCMPA.

Year	Viral Detection	n	%	*p*-Value ^1^
2015				
	CHIKV (+)	3	11.11	
	CHIKV (−)	11	40.74	0.0130 *
	ZIKV (+)	2	7.40	
	ZIKV (−)	11	40.74	
2016				
	CHIKV (+)	0	0	1.0000
	CHIKV (−)	2	50	
	ZIKV (+)	0	0	
	ZIKV (−)	2	50	
2017				
	CHIKV (+)	7	25	0.2940
	CHIKV (−)	10	35.71	
	ZIKV (+)	3	10.71	
	ZIKV (−)	8	28.57	

Source: Research protocol, 2022. Note: ^1^ Chi-square adherence (*p*-value < 0.05); * Significant values; CHIKV (+) = Positive detection for the CHIKV; ZIKV (+) = Positive detection for the ZIKV; CHIKV (−) = Negative detection for the CHIKV or ZIKV (−) = Negative detection for the ZIKV.

## Data Availability

The original contributions presented in this study are included in the article. Further inquiries can be directed to the corresponding author.

## References

[1] Oliveira M.T.S., Oliveira C.N.T., Marques L.M., Souza C.L., Oliveira M.V. (2020). Fatores associados ao aborto espontâneo: Uma revisão sistemática. Rev. Bras. Saúde Matern. Infant..

[2] Zahran A.B., Ali E.A., Siddeg W.A., Ali N.I., Bakheit K.H. (2016). Thyrotropin and Thyroid Antibodies in Sudanese Women with Recurrent Miscarriage. Khartoum Med. J..

[3] Zhou H., Liu Y., Liu L., Zhang M., Chen X., Qi Y. (2016). Maternal pre-pregnancy risk factors for miscarriage from a prevention perspective: A cohort study in China. Eur. J. Obs. Gynecol. Reprod. Biol..

[4] Chow S., Craig M., Jacques C., Hall B., Catteau J., Munro S., Scott G., Camaris C., McIver C., Rawlinson W. (2006). Correlates of placental infection with cytomegalovirus, parvovirus B19 or human herpes virus 7. J. Med. Virol..

[5] Zaki ME S., Goda H. (2007). Relevance of parvovirus B19, herpes simplex virus 2, and cytomegalovirus virologic markers in maternal serum for diagnosis of unexplained recurrent abortions. Arch. Pathol. Lab. Med..

[6] Kim I.D., Chang H.S., Hwang K.J. (2012). Herpes simplex virus 2 infection rate and necessity of screening during pregnancy: A clinical and seroepidemiologic study. Yonsei Med. J..

[7] Syridou G., Spanakis N., Konstantinidou A., Piperaki E., Kafetzis D., Patsouris E., Antsaklis A., Tsakris A. (2008). Detection of cytomegalovirus, parvovirus B19 and herpes simplex viruses in cases of intrauterine fetal death: Association with pathological findings. J. Med. Virol..

[8] Poletti M.O.D.O., Sousa C.F.S.S., Sampaio M.G. (2016). Evidências de transmissão vertical de arbovírus. Publicação Soc. Bras. Pediatr..

[9] Torres J.R., Falleiros-Arlant L.H., Dueñas L., Pleitez-Navarrete J., Salgado D.M., Brea-Del Castillo J. (2016). Congenital and perinatal complications of chikungunya fever: A Latin American experience. Int. J. Infect. Dis..

[10] Machain-Williams C., Machain-Williams C., Raga E., Raga E., Baak-Baak C.M., Baak-Baak C.M., Kiem S., Kiem S., Blitvich B.J., Blitvich B.J. (2018). Maternal, fetal, and neonatal outcomes in pregnant dengue patients in mexico. Biomed. Res. Int. N. Y..

[11] Cao B., Diamond M.S., Mysorekar I.U. (2017). Maternal-fetal transmission of zika virus: Routes and signals for infection. J. Interferon Cytokine Res. Auckl..

[12] Lopes N., Nozawa C., Linhares R.E.C. (2014). Características gerais e epidemiologia dos arbovírus emergentes no Brasil. Rev. Pan-Amaz. Saúde.

[13] Brasil Ministério da Saúde Secretaria de Vigilância em Saúde. Monitoramento dos Casos de Dengue, Febre de Chikungunya e Febre Pelo Vírus Zika até a Semana Epidemiológica 45. 2015, 46. https://www.saude.ba.gov.br/wp-content/uploads/2024/02/Protocolo-Tecnico-INVESTIGACAOBoletimpidemiológico-OBITO-ARBO.pdf.

[14] Rodriguez-Morales A.J., Cardona-Ospina J.A., Villamil-Gómez W., Paniz-Mondolfi A.E. (2015). How many patients with post-chikungunya chronic inflammatory rheumatism can we expect in the new endemic areas of Latin America?. Rheumatol. Int..

[15] Coronell-Rodriguez W., Arteta-Acosta C., Suárez-Fuentes M.A., Burgos-Rolon M.C., Rubio-Sotomayor M.T., Sarmiento-Gutierrez M., Corzo-Diaz C. (2016). Zika virus infection in pregnancy, fetal and neonatal impact. Rev. Chil. Infectol. Santiago.

[16] Rivadeneyra-Espinar P.G., Venegas-Esquivel G.A., Díaz-Espinoza C.M., Pérez-Robles V.M., González-Fernández M.I., Sesma-Medrano E. (2019). Zika como causa de aborto espontáneo en zonas endémicas. Boletín Médico Hosp. Infant. Méxic.

[17] Charlier C., Beaudoin M.C., Couderc T., Lortholary O., Lecuit M. (2017). Arboviruses and pregnancy: Maternal, fetal, and neonatal effects. Lancet Child Adolesc. Health Lond..

[18] Escobar M., Nieto A.J., Loaiza-Osorio S., Barona J.S., Rosso F. (2017). Pregnant women hospitalized with chikungunya virus infection, Colombia, 2015. Emerg Infect Dis. Foster City.

[19] Monteiro L.S., Cunha D.B., Sichieri R., Pereira R.A. (2018). Construção e Validação de Cartilha Educativa para Prevenção das Arboviroses na Gestação.

[20] Carlos A. (2017). Como Elaborar Projetos de Pesquisa.

[21] Freire M.C.M., Pattussi M.P., Estrela C. (2018). Tipos de estudos. Metodologia Científica. Ciência, Ensino e Pesquisa.

[22] Camargo LM A., Silva RP M., Meneguetti DU D.O. (2019). Research methodology topics: Cohort studies or prospective and retrospective cohort studies. J. Hum. Growth Dev..

[23] Araujo A.G., Aquino C.M.D.G.D., Souza D.Q.S.D., Ribeiro L.P., Jesus M.A.D.A. (2018). Aspectos Morfológicos Placentários na Gestação com Suspeita de Zika Vírus.

[24] Ayres M., Ayres M., Ayres D.L., Santos A.S. (2015). BioEstat 4.0: Aplicações Estatísticas nas Áreas das Ciências Biológicas e Médicas.

[25] Barbosa T., Ansaloni LV S., de Freitas A.A., de Carvalho R.L., Sousa T.B., de Oliveira R.A. (2021). A causalidade do abortamento espontâneo: Uma revisão integrativa. Braz. J. Health Rev. Curitiba.

[26] Laisk T., Soares A.L.G., Ferreira T., Painter J.N., Censin J.C., Laber S., Bacelis J., Chen C.-Y., Lepamets M., Lin K. (2020). The genetic architecture of sporadic and multiple consecutive miscarriage. Nat. Commun..

[27] Rodríguez Curcio H., Monsanto Hernández K., Colón J.A. (2016). Enfermedad trofoblastica gestacional diagnosticada en restos ovulares obtenidos de pacientes con abortos espontaneos. Rev. Obs. Ginecol. Venez..

[28] Rashid H., Ma E., Ferdous F., Ekström E.C., Wagatsuma Y. (2017). First-trimester fetal growth restriction and the occurrence of miscarriage in rural Bangladesh: A prospective cohort study. PLoS ONE.

[29] Cecatti J.G., Guerra G.V.D.Q.L., Sousa M.H.D., Menezes G.M.D.S. (2010). Aborto no Brasil: Um enfoque demografico. Rev. Bras. Ginecol. Obs..

[30] Xu G., Wu Y., Yang L., Yuan L., Guo H., Zhang F., Guan Y., Yao W. (2014). Risk factors for early miscarriage among Chinese: A hospital-based case-control study. Fertil. Steril..

[31] Correia L.L., Rocha H.A.L., Leite Á.J.M., Campos J.S., Silva A.C.E., Machado M.M.T., Rocha S.G.M.O., Gomes T.N., da Cunha A.J.L.A. (2018). Tendencia de abortos espontaneos e induzidos na região semiarida do Nordeste do Brasil: Uma serie transversal. Rev. Bras. Saude Mater. Infant..

[32] Alijotas-Reig J., Ferrer-Oliveras R., Rodrigo-Anoro Mj Farran-Codina I., Cabero-Roura L., Vilardell-Tarres M. (2010). Anti-β2-glycoprotein-I and anti-phosphatidylserine antibodies in women with spontaneous pregnancy loss. Fertil. Steril..

[33] Dos Santos Durex É.W., Vieira Dias F., Braga Rodrigues R., Midlej Neto T.K., Valente Rocha L.L. (2016). Abortos Espontâneos No Brasil E Em Suas Regiões: Estudo De Prevalência. Braz. J. Surg. Clin. Res..

[34] Soares A.M., Cançado F.M.A.A. (2018). Perfil De Mulheres Com Perda Gestacional. Rev. Med. Minas. Gerais.

[35] Chaves J.H.B., Oliveira E.M., Bezerra A.F.S., Camano L., Sun S.Y., Mattar R. (2011). O abortamento incompleto (provocado e espontâneo) em pacientes atendidas em maternidade do Sistema Único de Saúde. Rev Bras Clin Med..

[36] Buss L., Tolstrup J., Munk C., Bergholt T., Ottesen B., Grønbæk M., Kjaer S. (2006). Spontaneous abortion: A prospective cohort study of younger women from the general population in Denmark. Validation, occurrence and risk determinants. Acta Obstet. Et Gynecol..

[37] Rosadas C., Brites C., Arakaki-Sánchez D., Casseb J., Ishak R. (2021). Protocolo Brasileiro para Infecções Sexualmente Transmissíveis 2020: Infecção pelo vírus Zika. Epidemiol. Serv. Saúde.

[38] Kac G., Silveira E.A., Oliveira L.C.D., Araújo D.M.R., Sousa E.B.D. (2007). Fatores associados à ocorrência de cesárea e aborto em mulheres selecionadas em um centro de saúde no município do Rio de Janeiro, Brasil. Rev. Bras. Saúde Matern. Infant..

[39] de Araújo C.P., de Rezende Dornelas A.C.V., Sousa A.M. (2018). Abordagem terapêutica no processo de esvaziamento uterino. Rev. Baiana Enferm..

[40] Arcanjo F.C.N., Ribeiro A.S., Teles T.G., Macena R.H.M., Carvalho F.H.C. (2011). Uso do misoprostol em substituição à curetagem uterina em gestações interrompidas precocemente. Rev. Bras. Ginecol. Obstet..

[41] Secretaria de Vigilância em Saúde, Secretaria de Atenção a Saúde (2013). ZIKA: Abordagem na Atenção Básica.

[42] Nanda K., Peloggia A., Grimes D., Lopez L., Nanda G. (2012). Expectant care versus surgical treatment for miscarriage. Cochrane Database Syst. Rev..

[43] Morris J.L., Winikoff B., Dabash R., Weeks A., Faundes A., Gemzell-Danielsson K., Visser G.H.A. (2017). Recomendações atualizadas da FIGO para misoprostol utilizadas isoladamente em ginecologia e obstetrícia. Rev. Int. De. Ginecol. Obs..

[44] Camayo F.J.A., Martins L.A.B., Cavalli R.D.C. (2011). Perda gestacional retida: Tratamento baseado em evidência. Femina.

[45] Bombin M., Mercado J., Zúñiga J., Encalada D., Ávila J. (2019). Aspiración manual endouterina (AMEU): Revisión de la literatura y estudio de serie de casos. Rev. Chil. Obs. Ginecol..

[46] Nunes P., Nogueira R., Coelho J., Rodrigues F., Salomumasobre, Joséé C., De Carvalho J., Rabelo K., De Azeredo E., Baseulio-Deoliveira R. (2019). Investigação de múltiplos órgãos natimortos de uma infecção materna por denv-4: Caracterização histopatológica e de mediadores inflamatórios. Víruses.

[47] Mineiro J., Cao B., Govero J., Smith A., Fernandez E., Cabrera O., Garber C., Noll M., Klein R., Noguchi K. (2016). A infecção pelo vírus Zika durante a gravidez em camundongos causa danos à placenta e morte fetal. Célula.

[48] Gregory C.J., Oduyebo T., Brault A.C., Brooks J.T., Chung K., Hills S., Kuehnert M.J., Mead P., Meaney-Delman D., Rabe I. (2017). Modos de transmissão do vírus Zika. J. Infect. Dis..

[49] Gérardin P., Barau G., Michael A., Bintner M., Randrianaivo H., Choker G., Lenglet Y., Touret Y., Bouveret A., Grivard P. (2008). Estudo prospectivo multidisciplinar de infecções materno-infantis pelo vírus Chikungunya na Ilha de La Ré União. PLoS Med..

[50] Touret Y., Randrianaivo H., Michael A., Schuffenecker I., Kauffmann E., Lenglet Y., Barau G., Fourmaintraux A. (2006). Transmissão materno-fetal precoce do vírus Chikungunya. Press. Médica.

[51] Grivard P., Le Roux K., Laurent P., Fianu A., Perrau J., Gigan J., Hoarau G., Grondin N., Staikowsky F., Favier F. (2007). Diagnóstico molecular e sorológico da infecção pelo vírus Chikungunya. Patol. Biol..

[52] Prata-Barbosa A., Cleto-Yamane T., Robaina J., Guastavino A., De Magalhumaes-Barbosa M., Brindeiro R., Medronho R., Da Cunha A. (2018). Co-infecção por vírus Zika e Chikungunya associada à morte fetal—Relato de caso. Int. J. Infectar. Des..

[53] Imbeloni A.A. (2021). Infecção Experimental Por Vírus Zika Em Fêmeas Prenhes De Saimiri Collinsi: Avaliação No Pré-natal E Síndrome Congênita Do Zika Vírus. https://bdtd.ibict.br/vufind/Record/IEC-2_a0927a43c5bfa6b4b44a413097d56e4b.

[54] van der Eijk A.A., van Genderen P.J., Verdijk R.M., Reusken C.B., Mögling R., van Kampen J.J.A., Widagdo W., Aron G.I., GeurtsvanKessel C.H., Pas S.D. (2022). Miscarriage Associated with Zika Virus Infection. N. Engl. J. Med..

[55] Runge-Ranzinger S., Morrison Ac Manrique-Saide P., Horstick O. (2019). Padrões de transmissão do Zika: Uma metarevisão. Trop. Med. Int. Heal..

[56] Salomão N., Brendolin M., Rabelo K., Wakimoto M., De Filippis A., Dos Santos F., Moreira M., Basílio-De-Oliveira C., Avvad-Portari E., Paes M. (2021). Aborto Espontâneo e Infecção por Chikungunya: Achados Patológicos. Rev. Vírus.

[57] Yu W., Hu X., Cao B. (2022). Viral Infections During Pregnancy: The Big Challenge Threatening Maternal and Fetal Health. Matern. Fetal Med..

[58] Nwabuobi C., Arlier S., Schatz F., Guzeloglu-Kayisli O., Lockwood C., Kayisli U.A. (2017). hcg: Funções biológicas e aplicações clínicas. Int. J. Mol. Sci..

[59] Martines R.B., Bhatnagar J., de Oliveira Ramos A.M., Davi H.P.F., Iglezias A., Kanamura C.T., Keating M.K., Hale G., Silva-Flannery L., Muehlenbachs A. (2016). Pathology of congenital Zika syndrome in Brazil: A case series. Lancet.

[60] Dulay A.T. (2020). Manual MSD. Aborto Espontâneo. https://www.msdmanuals.com/pt/casa/problemas-de-saúde-feminina/complicações-da-gravidez/trabalho-de-parto-prematuro?query=dulay.

[61] Mehrjardi M., Shobeirian F. (2017). O papel da placenta na infecção pré-natal pelo vírus Zika. Doença Vírus..

[62] Azevedo R.S.S., Araujo M.T., Oliveira C.S., Martins Filho A.J., Nunes B.T.D., Henriques D.F., Silva E.V.P., Carvalho V.L., Chiang J.O., Martins L.C. (2018). Zika Virus Epidemic in Brazil. II. Post-Mortem Analyses of Neonates with Microcephaly, Stillbirths, and Miscarriage. J. Clin. Med..

[63] Contopoulos-Ioannidis D., Newman-Linsay S., Chow C., Labeaud A. (2018). Transmissão de mãe para filho do vírus Chikungunya; a Revisão sistemática e metanálise. PloS Negl. Trop. Dis..

[64] Villamil-Gómez W., González-Camargo O., Rodriguez-Ayubi J., Zapata-Serpa D., Rodriguez-Morales A. (2016). Co-infecção de dengue, chikungunya e zika em paciente da Colômbia. J. Infectar Saúde Pública.

[65] Mercado M., Acosta-Reyes J., Parra E., Pardo L., Rico A., Campo A., Navarro E., Viasus D. (2016). Características clínicas e histopatológicas de casos fatais com coinfecção pelo vírus dengue e chikungunya na Colômbia, 2014 a 2015. Eurosurveillance.

